# A survey of allergic conjunctivitis in children in China

**DOI:** 10.1038/s41598-022-25591-7

**Published:** 2022-12-05

**Authors:** Xu Gao, Lan Hong, Qin Xiang

**Affiliations:** 1 Department of Ophthalmology, Bi Shan Hospital of Chongqing, Chongqing, 402700 China; 2 Department of Ophthalmology, Children’s Hospital of Chongqing Medical University, National Clinical Research Center for Child Health and Disorders, Ministry of Education Key Laboratory of Child Development and Disorders, Chongqing, 400014 China; 3 China International Science and Technology Cooperation Basis of Child Development and Critical Disorders, Chongqing, China; 4 Chongqing Engineering Research Center of Stem Cell Therapy, Chongqing, China

**Keywords:** Health care, Risk factors, Signs and symptoms

## Abstract

To explore the risk factors for allergic conjunctivitis (AC) in Chinese children. We recruited 176 children who suffered from AC and a control group comprising 131 normal subjects in southern China. Each participant completed a questionnaire and underwent multiple eye examinations and a skin prick test (SPT). The data of the questionnaire, the scores of the symptoms/signs and the results of the SPT were analysed. The rate of parental allergic history in the case group was much higher than that in the control group (P < 0.01). Compared with the control group, the case group was more likely to have other concomitant diseases (P < 0.01). The scores of ocular symptoms/signs had a significant correlation with the clinical duration of AC in the case group (P < 0.01). Children with other concomitant diseases or a parental allergic history were more likely to have AC.

## Introduction

Allergic conjunctivitis (AC) is a group of diseases associated with type I (IgE-mediated hypersensitivity) and type IV hypersensitivity reactions (non-IgE-mediated hypersensitivity). It is considered a hypersensitivity disorder of the ocular surface, and its symptoms are often, but not always, associated with other concomitant diseases, such as rhinitis and asthma^1,2^. AC includes seasonal allergic conjunctivitis (SAC), perennial allergic conjunctivitis (PAC), vernal keratoconjunctivitis (VKC), atopic keratoconjunctivitis (AKC), and giant papillary conjunctivitis (GPC). SAC and PAC comprise the majority of ocular allergic conditions, whereas severe conditions, such as AKC and VKC, affect only a small group of patients^3^. The main subjective symptoms in AC are itching, redness of the eyes and photophobia, while objective signs include chemosis, bulbar conjunctival hyperaemia and mucus secretion. Until now, little attention has been given to AC, and AC has generally been treated as a comorbid condition with other atopic disorders, such as asthma and allergic rhinitis (AR); therefore, our understanding of AC is less than that for AR and asthma^4–9^. In addition, current studies on AC mainly focus on adults or elderly children, and investigations of AC in children are very scarce; therefore, relevant data are lacking^10–12^.

In a previous report, house dust mites were the most prevalent allergen in patients with asthma and/or rhinitis in China^13–16^. *Dermatophagoides farinae* and *Dermatophagoides pteronyssinus* are the dominant species in our country^17^ and are also common sensitising agents in AC^18,19^. Allergy to dust mites is considered to be a risk factor for AC. The skin prick test (SPT) is more specific and sensitive than total IgE levels and has been widely used as a supplemental tool for the diagnosis of AC, AR and asthma^20,21^. The signs and symptoms of AC are not very typical in children. In clinical practice, children are often misdiagnosed or never diagnosed because of the variety of atypical manifestations and the lack of typical signs, and they may be too young to express their feelings accurately. Many children were diagnosed with hyperactivity or Tourette syndrome before they visited the ophthalmology department.

In this study, we aimed to explore the risk factors for AC in Chinese children and expect to provide helpful advice about treatments in the future.

## Results

### Clinical features

AC morbidity was higher in male than in female children (P < 0.01) (Table 1). There was no significant difference in age between the control group and the case group.

**Table 1:** The risk factors related to allergic conjunctivitis.

	–	Control group	Case group	P value
Age	Mean ± SD	5.92 ± 2.51	6.21 ± 2.74	0.34
	M (Q1–Q3)	6.00 (4.08–8.17)	5.83 (4.00–7.42)	
Sex	Female	70 (53.44%)	46 (26.14%)	< 0.01
	Male	61 (46.56%)	130 (73.86%)	
Father	0*	127 (96.95%)	107 (60.80%)	< 0.01
	1**	4 (3.05%)	69 (39.20%)	
Mother	0*	129 (98.47%)	97 (55.11%)	< 0.01
	1**	2 (1.53%)	79 (44.89%)	
Both	0*	131 (100%)	173 (98.30%)	0.26
	1**	0 (0%)	3 (1.70%)	

*means without allergic history.

**means with allergic history.

### Parental history of allergic disease

The questionnaire also collected information about the parental history of allergic diseases, including AR, asthma, eczema, urticaria and atopic dermatitis. The rate of parental allergy history in the case group was much higher than that in the control group (P < 0.01) (Table 1). AR was the most common allergic disease in parents, with 18.69% of fathers and 24.18% of mothers reporting an AR history. In addition, in 8 children in the case group, both parents had a history of AR (data not shown).

### Bedtime routines

The correlation between sleep style and the severity of symptoms/signs was investigated. The results showed that an earlier bedtime was associated with lower scores of ocular symptoms/signs (P < 0.01). Longer sleep hours were associated with lower scores of ocular symptoms/signs (P < 0.05) (Table 2).

**Table 2:** The correlations between scores of ocular symptoms/signs and style of sleep.

		Sign scores	Symptom scores	Mean ± SD
Bedtime time	*r*s	0.39	0.37	9.51 pm (8:53–10:49 pm)
	P value	0.001	0.003	
Total sleep hours	*r*s	− 0.26	− 0.27	8.12 ± 0.86 (hours)
	P value	− 0.04	0.03	

### AC accompanied by other concomitant diseases

We further studied the relationship between other concomitant diseases and AC in children using the questionnaire. In the case group, the predominant condition was AR, at 85.80%, followed by eczema (76.14%) and asthma (65.34%). Atopic dermatitis and urticaria papulosa were also more common in the case group than in the control group. Compared with those in the control group, these findings were all significantly different (all P < 0.01) (Table 3).

**Table 3:** The subjects with other concomitant diseases in two groups.

		Control group	Case group	P value
Allergic rhinitis	0	126 (96.18%)	25 (14.20%)	< 0.01
	1	5 (3.82%)	151 (85.80%)	
Asthma	0	122 (93.13%)	61 (34.66%)	< 0.01
	1	9 (6.87%)	115 (65.34%)	
Atopic dermatitis	0	131 (100.00%)	141 (80.11%)	< 0.01
	1	0 (0.00%)	35 (19.89%)	
Urticaria papulosa	0	122 (92.86%)	97 (55.56%)	< 0.01
	1	9 (7.14%)	79 (44.44%)	
Eczema	0	116 (88.55%)	42 (23.86%)	< 0.01
	1	15 (11.45%)	134 (76.14%)	
Adenoidal hypertrophy	0	129 (98.47%)	165 (93.75%)	0.048
	1	2 (1.53%)	11 (6.25%)	

0 means without this disease.

1 means with this disease.

### Eye symptoms

**Figure 1:**
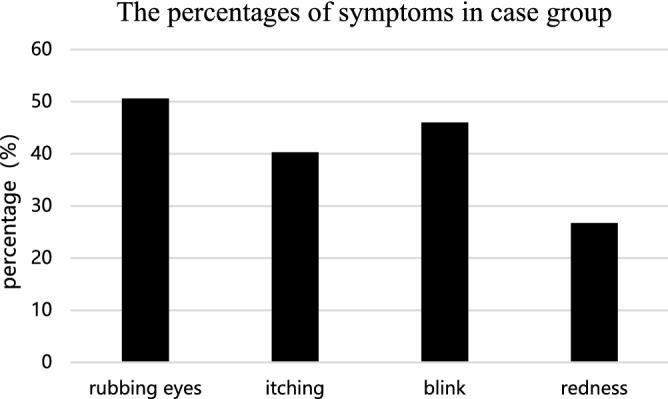
The percentages of symptoms in case group. 50.6% of AC patients had rubbing eyes (n = 89); 40.3% had itching (n = 71); 46.0% had blinking (n = 81). 26.7% had redness (n = 47).

The top three symptoms of AC were eye rubbing, itching, and blinking. More than half of the children in the case group suffered from these symptoms. Redness was the fourth most common complication, with a rate of 26.7% in the case group, although it was hard to distinguish from redness due to other types of conjunctivitis (Fig. 1).

### Ocular signs

**Figure 2:**
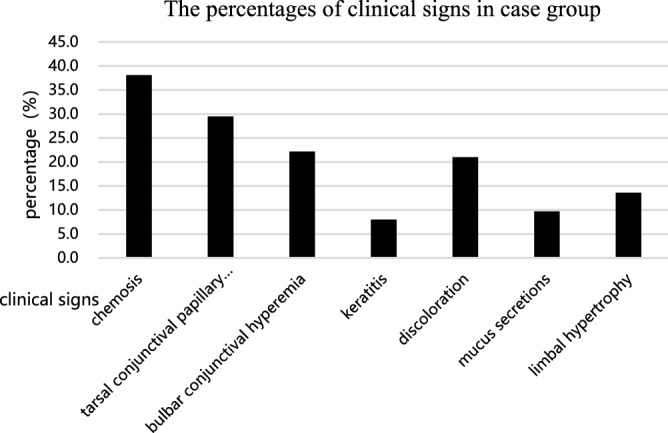
The percentages of clinical signs in case group. 38.1% of AC patients had chemosis (n = 67); 29.5% had tarsal conjunctival papillary hypertrophy (n = 52); 22.2% had bulbar conjunctival hyperemia (n = 39); 8.0% had keratitis (n = 14); 21.0% had discoloration (n = 37); 9.7% had mucus secretions (n = 17); 13.6% patients had limbal hypertrophy (n = 24).

In the case group, the top three clinical signs of AC were chemosis, tarsal conjunctival papillary hypertrophy and bulbar conjunctival hyperaemia; however, none of them occurred in more than half of the subjects. Keratitis was uncommon, with a proportion of 8.0%, which was consistent with previous reports, as corneal involvement rarely occurs in AC^22^ (Fig. 2). However, keratitis is transient and is mostly caused by mechanical rubbing of the eye. After the application of timely anti-allergic treatment, corneal epithelial irritation can be relieved quickly. Moreover, only 2.84% (n = 5) of the patients had scales or scurf on the eyelid skin, and 2.27% (n = 4) had meibomian gland obstruction (data not shown).

### Relationship between ocular symptoms/sign scores and the duration of AC

The mean clinical duration of AC in the case group was 3.09 ± 2.92 months. We found significant correlations between the scores of ocular symptoms/signs and the duration of AC (P < 0.01) (Table 4).

**Table 4:** Relationship between ocular symptoms/sign scores and the duration of AC.

		Clinical course (months)	Mean ± SD
Sign scores	*r*s	0.81	4.91 ± 3.73
	P value	< 0.01	
Symptom scores	*r*s	0.85	7.49 ± 4.66
	P value	< 0.01	

### Relationship between scores of ocular symptoms/signs and SPT results

The results of the SPT in the control group were all negative. In our study, we found that mites were the most common allergen (*D. pteronyssinus* 80.7% and *D. farina* 76.1%), followed by pollen (15.4%) and pets (6.5%) (data not shown). In the case group, the results of skin reactions to *D. pteronyssinus* were in accordance with the *D. farina* results; the goodness of fit reached 81.25% (n = 143), and grade 3 was the main degree of SPT results induced by these two allergens (Table 5).

**Table 5:** Grades of the SPT results in case group.

	Grades	N	%
*Dermatophagoides pteronyssinus*	Grade 1	34	19.32%
	Grade 2	38	21.59%
	Grade 3	70	39.77%
	Grade 4	34	19.32%
*Dermatophagoides farinae*	Grade 1	42	23.86%
	Grade 2	24	13.64%
	Grade 3	76	43.18%
	Grade 4	34	19.32%

Grade 1 is 25% of area of histamine-induced wheal, Grade 2 is 50% of this area, Grade 3 is 100% of this area, Grade 4 is 200% of it.

We further investigated the relationships between the scores of ocular symptoms/signs and the results of SPTs related to these two dust mite allergens but failed to find any correlation. The data are shown in Supplementary Table 1.

## Discussion

In this study, we analysed AC in children who were diagnosed with SAC or PAC since these two diseases make up the majority of AC diseases. In addition, they have the same signs and symptoms, and they affect a patient’s quality of life^2^.

We found that some AC patients also had transient keratitis that appeared to be different from AKC or VKC. This type of keratitis presented as superficial punctate keratitis rather than inflammation. Children cannot always control rubbing their eyes because of itchiness, which could lead to a mechanical abrasion of the cornea.

In this study, we found that AC patients had more frequent and severe allergic reactions to house dust mites, and mites may be the most common allergen in children with AC, which was consistent with a previous report in children^10^. Although no correlations were found between the scores of ocular symptoms/signs and the results of the SPT in response to dust mite allergens, our results indicated a significantly positive correlation between the scores and duration of AC. The longer the disease was sustained, the higher the scores of symptoms/signs the patients received. We presumed that a persistent allergic reaction, whether moderate or severe, to dust mite allergens may be responsible for the ocular symptoms and signs. Therefore, to avoid failure treatment and prevent the aggravation of symptoms and signs caused by delayed diagnosis, timely anti-allergic treatment is recommended.

We observed in this study that more males had AC than females, and males had a higher risk of developing AC than females, which was consistent with other studies^10,23–25^, however, the reasons for this difference remain unknown. They could be due to different lifestyles or genetic susceptibilities and need further study.

We found that parental allergic history was a risk factor for AC in children, and AR was the most common allergic disease among parents.

This study showed that an earlier bedtime and longer sleep hours were both associated with lower scores of ocular symptoms/signs. This may suggest that adequate sleep hours in children can help protect against allergic reactions, possibly due to a well-working immune system.

We found that less than half of the subjects had ocular signs caused by AC; this result was different from that in adults. In children, sometimes obvious signs are lacking, so they are more likely to be misdiagnosed or never diagnosed. Therefore, inquiring about the allergic history, parental allergic history, and season of onset and performing a SPT if possible, will help to definitively diagnose AC.

This study had some limitations. First, to the best of our knowledge, this is the first study on AC in children of a young age, but the sample size in this study is small. In future research, we need to recruit more children to verify our findings. Second, we used the SPT instead of the conjunctival provocation test, which is an established diagnostic procedure for AC, because the conjunctival provocation test is not usually used in the clinic due to the relative risks in children^26^. The SPT has a higher diagnostic accuracy than serum-specific IgE in vitro^3,27^. Despite this, the conjunctival provocation test, serum-specific IgE detection, and SPT can be considered tools for evaluating only the allergic status of individuals and not the final diagnosis^28^. Since our subjects were too young to complete the questionnaires adequately, all the questionnaires were completed by their parents, but parents may have limited understanding of their child’s routines. Additionally, although some studies showed a relationship between allergic diseases and sleep^29^, we failed to find a correction. In some studies, there were associations between AR and poor sleep parameters, including sleep latency, sleep apnoea and daytime dysfunction^30^.

## Conclusion

In this study, we explored some risk factors for AC in children. The results showed that concomitant diseases and a family allergy history were closely related to the incidence of AC in children.

## Methods

This study included of a disease-specific questionnaire, an ophthalmologic examination and a SPT.

The study included 176 subjects suffering from AC and 131 normal control subjects in the Children’s Hospital of Chongqing Medical University (Chongqing, China) from 2016 to 2019. All subjects were aged from 3 to 9 years. Informed consent was obtained from both parents. The SPT was performed in all subjects, including those in the control group and case group.

In this study, we enrolled AC patients diagnosed with SAC or PAC since these two diseases make up the majority of AC diseases, are very common in children and have the same signs and symptoms^2^.

The diagnosis of ocular allergy was based on the clinical history, signs and symptoms, and a positive SPT result. Signs included chemosis, papillae, bulbar conjunctival hyperaemia, discolouration, discolouration and mucus secretion. Symptoms included eye rubbing, itching, blinking and redness.

Since the subjects were too young to respond adequately to the questionnaire, all the questionnaires were answered by the children’s parents. The questionnaire collected basic demographic data, symptoms and signs, whether they had systemic allergic diseases, the onset season of AC, and parental history of allergic diseases. Our ophthalmologists performed several eye exams, including a primary ophthalmologic examination, slit lamp observation, and corneal fluorescent staining. The corneal fluorescein staining scores were modified according to the National Eye Institute grading scale^13^. The measurements to obtain scores of symptoms and signs were adapted from Macy M. S. Wu et al. (Supplementary Table 2)^31,32^.

The SPTs were performed by using commercial extracts of 13 common inhalation allergen*s: D. farinae, D. pteronyssinus,* cockroaches, Saccharomycetes, Penicillium, dog fur, cat hair, duck feathers, birch pollen, Artemisia pollen, maize pollen, cotton wool and cigarettes. The negative control solution was a phenolated glycerol-saline solution. The positive control solution was 10 mg/ml histamine hydrochloride (ALK- ABELLO Laboratories, Holmolm, Denmark). All patients enrolled in the study had discontinued antiallergic drugs for at least 10 days prior to the SPT. All the doctors who performed the SPTs were well trained and had already obtained a professional certificate.

Skin reactions were assessed 20 min later. Skin test positivity was defined as an allergen induration diameter 3 mm larger than the negative control diameter. The skin index was graded from 0 to 4 by comparing the size with positive and negative controls^33^. Grade 0 was defined as no wheal appearance. Grade 1 was defined as 25% of the area compared with the histamine-induced wheal, Grade 2 was defined as 50%, Grade 3 was defined as 100%, and Grade 4 was defined as 200%. Grades 1 to 4 were considered positive skin reactions^34^.

The intergroup indicator parameters of the case and control groups were described and compared. Quantitative data are described as the mean ± standard deviation (SD) and were compared by the t test. Rank data are described as the median (quartile spacing), and rank sum Wilcoxon or Kruskal–Wallis tests were used for intergroup comparisons. Qualitative data are described as the frequency (percentage), and the chi-square test was used to compare the two groups. All P values are two-sided. A P value of 0.05 was considered statistically significant. The confidence interval was 95%. SPSS software (version 21.0) was used for all statistical calculations (SPSS, Inc., Chicago, IL, USA).

### Ethics approval and consent to participate

The study followed the Tenets of the Declaration of Helsinki and was approved by the Ethics Committee of the Children’s Hospital of Chongqing Medical University, Chongqing, China (Permit No. 004/2016). Written informed consent was obtained from the parents of each subject.

## Supplementary Information

Supplementary Table 1.

Supplementary Table 2.

Supplementary Information 3.

## Data Availability

The data that support the findings of this study are available from Children’s Hospital of Chongqing Medical University but restrictions apply to the availability of these data, which were used under license for the current study, and so are not publicly available. Data are however available from the authors upon reasonable request and with permission of Children’s Hospital of Chongqing Medical University.
