# Supplementary material for: A survey of allergic conjunctivitis in children in China
Source: Sci Rep. 2022 Dec 5;12:21026. doi: 10.1038/s41598-022-25591-7 (PMC9722788; doi:10.1038/s41598-022-25591-7)
Supplement: Supplementary file 1 — Supplementary Table 1. [file 41598_2022_25591_MOESM1_ESM.docx]

| House dust mites | | | Grading of SPT results | | | | | total | P value | |
| --- | --- | --- | --- | --- | --- | --- | --- | --- | --- | --- |
|  |  |  | 0 | 1 | 2 | 3 | 4 |  |  |  |
| DP^*^ | Symptom score | Mean±SD | 1.00±0.92 | 1.53±1.06 | 1.44±0.96 | 1.47±0.90 | 1.54±1.27 | 1.38±1.00 | | 0.46 |
|  |  | M(Q1～Q3) | 1.00(0.00～2.00) | 1.00(1.00～2.00) | 1.00(1.00～2.00) | 1.00(1.00～2.00) | 1.00(1.00～3.00) | 1.00(1.00～2.00) | |  |
|  |  | Min～Max | 0.00～3.00 | 0.00～4.00 | 0.00～3.00 | 0.00～3.00 | 0.00～3.00 | 0.00～4.00 | |  |
|  | Sign score | Mean±SD | 3.55±2.06 | 3.00±2.78 | 3.82±1.91 | 4.09±2.33 | 3.64±1.63 | 3.70±2.21 | | 0.59 |
|  |  | M(Q1～Q3) | 4.00(2.00～5.00) | 3.00(0.50～4.50) | 3.00(3.00～4.00) | 4.00(2.00～5.50) | 3.00(3.00～4.00) | 3.00(2.00～5.00) | |  |
|  |  | Min～Max | 0.00～7.00 | 0.00～10.00 | 1.00～9.00 | 1.00～9.00 | 1.00～7.00 | 0.00～10.00 | |  |
| DF^**^ | Symptom score | Mean±SD | 0.40±0.89 | 1.63±1.16 | 1.36±0.92 | 1.58±0.97 | 1.62±1.26 | 1.49±1.07 | | 0.20 |
|  |  | M(Q1～Q3) | 0.00(0.00～0.00) | 1.00(1.00～3.00) | 1.00(1.00～2.00) | 1.00(1.00～2.00) | 1.00(1.00～3.00) | 1.00(1.00～2.00) | |  |
|  |  | Min～Max | 0.00～2.00 | 0.00～4.00 | 0.00～3.00 | 0.00～3.00 | 0.00～3.00 | 0.00～4.00 | |  |
|  | Sign score | Mean±SD | 2.20±1.92 | 3.45±2.74 | 4.90±2.85 | 3.68±1.75 | 3.64±1.78 | 3.67±2.21 | | 0.43 |
|  |  | M(Q1～Q3) | 2.00(1.00～3.00) | 3.00(1.50～4.50) | 4.50(2.00～7.00) | 3.00(3.00～4.00) | 4.00(3.00～4.00) | 3.00(2.00～4.00) | |  |
|  |  | Min～Max | 0.00～5.00 | 0.00～10.00 | 2.00～9.00 | 1.00～9.00 | 1.00～7.00 | 0.00～10.00 | |  |

**Supplementary table 1. The correlation between the scores of ocular symptoms/signs and the grades of SPT results responding to these two dust mites**

DP^*^: dermatophagoides pteronyssinus

DF^**^: dermatophagoides farinae
