# Supplementary material for: A survey of allergic conjunctivitis in children in China
Source: Sci Rep. 2022 Dec 5;12:21026. doi: 10.1038/s41598-022-25591-7 (PMC9722788; doi:10.1038/s41598-022-25591-7)
Supplement: Supplementary file 2 — Supplementary Table 2. [file 41598_2022_25591_MOESM2_ESM.docx]

**Supplementary table 2. Scores of symptoms and signs of AC.**

| Item | Scores | | | |
| --- | --- | --- | --- | --- |
|  | 0 | 1 | 2 | 3 |
| *symptoms* |  |  |  |  |
| Rubbing eyes | No | mild | moderate | severe |
| Itching | No | occasional | frequent | constant |
| Blink | No | occasional | frequent | constant |
| Redness | No | mild | moderate | severe |
| *signs* |  |  |  |  |
| Chemosis | No | one quadrant | two to three quadrants | four quadrants |
| Tarsal conjunctival papillary hypertrophy | No | mild | moderate, <1/3 palpebral conjunctival area | severe, appear visualization of the deep tarsal vessels |
| Bulbar conjunctival hyperemia | No | mild | moderate | severe |
| Keratitis | No | one quadrant | two quadrants | three or four quadrants |
| Discoloration | No | mild | moderate | severe |
| Mucus secretions | No | small amount | moderate amount | eyelid was glued in morning |
| Limbal hypertrophy | No | one quadrant | two to three quadrants | more than three quadrants |
