# Supplementary material for: A survey of allergic conjunctivitis in children in China
Source: Sci Rep. 2022 Dec 5;12:21026. doi: 10.1038/s41598-022-25591-7 (PMC9722788; doi:10.1038/s41598-022-25591-7)
Supplement: Supplementary file 3 — Supplementary Information 3. [file 41598_2022_25591_MOESM3_ESM.docx]

**Questionnaire**

**Basic information**

| Name |  | Gender: | □male □female | Age |  |
| --- | --- | --- | --- | --- | --- |
| ID |  | Telephone number | | | |

**Questionnaire section**

| Course of disease: months | |
| --- | --- |
| Feeding history | □Artificial/ □ Mixing/ □Exclusive breast feeding(Duration: months ) |
| Food supplement | months, If add the following food for the first time: □eggs □ seafood |
| Bedtime routines | pm |
| [Immunity](javascript:;) | catch colds frequently □ yes □ no |
| Living environment | □Urban area □ District and county □ Rural area |
| Systemic allergic disease | □ Allergic rhinitis □ Allergic asthma □Papular urticaria □ Eczema  □ Atopic dermatitis □ Adenoid hyperplasia |
| Family history: | Father: □Allergic conjunctivitis □Allergic rhinitis □ Asthma □ Urticaria □Neurodermatitis □Eczema  Mother: □Allergic conjunctivitis □Allergic rhinitis □ Asthma □ Urticaria □Neurodermatitis □Eczema |

**Ophthalmic symptom section**

| - □Itching □BLinking □Rubbings eyes □Redness □Chemosis □Photophobia □[Lacrimation](javascript:;) □Under-eye dark circle □Madaroses - Frequency of attacks : times per year; - Attack season: □Spring □Summer □Autumn □Winter □Late-spring/early summer □Late autumn and early winter |
| --- |

**Physical examination section**

| - Palpebral conjunctiva: □Congestion and vascular blur □Pallid conjunctiva Eyelid marginal hyperemia - □Eyelid conjunctival edema □ Follicles □ Papillary conjunctiva - Meibomian: □The opening of the meibomian gland is obstructed - Bulbous conjunctiva: □Hyperemia □ Edema discoloration □ Corneal limbal hypertrophy - Superficial punctate keratitis: scores; - Eyelid skin: □ Eye pigmentation (Dark circles) □ Allergic eyelid dermatitis - Secretions: □ Mucus □Watery □ Purulent - Breakup time of tear film(BUT): □ right eye □left eye |
| --- |

**The results of SPT**

| Allergen | Result | Allergen | Result | Allergen | Result |
| --- | --- | --- | --- | --- | --- |
| Dermatophagoides pteronyssinus |  | Cockroaches |  | Duck feather |  |
| Cotton wool |  | [Cigarette](javascript:;) |  | [Saccharomycetes](javascript:;) |  |
| [Dog Fur](javascript:;) |  | Dematophagoides farinae |  | [Penicillium](javascript:;) |  |
| Cat hair |  | Artemisia pollen |  | 0.01mg/ml histamine hydrochloride |  |
| Birch pollen |  | Maize pollen |  | Saline solution |  |
